# New horizons in the ageing autonomic nervous system: orthostatic hypotension and supine hypertension

**DOI:** 10.1093/ageing/afac150

**Published:** 2022-08-02

**Authors:** Melanie Dani, Patricia Taraborrelli, Dimitrios Panagopoulos, Andreas Dirksen, Miriam Torocastro, Richard Sutton, Phang Boon Lim

**Affiliations:** Imperial Syncope Unit, Imperial College Healthcare NHS Trust, London W12 0HS, UK; Cutrale Peri-operative and Ageing group, Department of Bioengineering, Imperial College London, London W12 0BZ, UK; Imperial Syncope Unit, Imperial College Healthcare NHS Trust, London W12 0HS, UK; Imperial Syncope Unit, Imperial College Healthcare NHS Trust, London W12 0HS, UK; Imperial Syncope Unit, Imperial College Healthcare NHS Trust, London W12 0HS, UK; Imperial Syncope Unit, Imperial College Healthcare NHS Trust, London W12 0HS, UK; National Heart and Lung Institute, Imperial College London, London, UK; Imperial Syncope Unit, Imperial College Healthcare NHS Trust, London W12 0HS, UK

**Keywords:** autonomic nervous system, baroreflex, sympathetic, parasympathetic, hypertension, orthostatic hypotension, older people

## Abstract

Blood pressure regulation is an automatic, moment-by-moment buffering of the blood pressure in response to physiological changes such as orthostasis, exercise and haemorrhage. This finely orchestrated reflex is called the baroreflex. It is a regulated arc of afferent, central and efferent arms. Multiple physiological changes occur with ageing that can disrupt this reflex, making blood pressure regulation less effective. In addition, multiple changes can occur with ageing-related diseases such as neurodegeneration, atherosclerosis, deconditioning and polypharmacy. These changes commonly result in orthostatic hypotension, hypertension or both, and are consistently associated with multiple adverse outcomes. In this article, we discuss the healthy baroreflex, and physiological and pathophysiological reasons for impaired baroreflex function in older people. We discuss why the common clinical manifestations of orthostatic hypotension and concomitant supine hypertension occur, and strategies for balancing these conflicting priorities. Finally, we discuss strategies for treating them, outlining our practice alongside consensus and expert guidance.

## Key Points

- The healthy baroreflex regulates moment-by-moment buffering of blood pressure, responding to physiological changes.
- Multiple physiological and pathophysiological changes occur with ageing that impair the sensitivity of the baroreflex.
- An impaired baroreflex means impaired blood pressure regulation—often resulting in orthostatic hypotension and hypertension.
- Orthostatic hypotension is consistently associated with adverse cardiovascular outcomes.
- Concomitant orthostatic hypotension and supine hypertension commonly co-occur and require careful management

## Introduction

Impaired blood pressure (BP) regulation and its consequences are common in older patients. Orthostatic hypotension (OH) in particular is responsible for almost a quarter of Emergency Department attendances with syncope [1]. Defined as a fall in 20 mmHg or more of systolic BP or 10 mmHg or more of diastolic BP within 3 min of standing or head-up tilt [2], it is consistently associated with falls, injury [3, 4], an increased risk of adverse cardiac events and all-cause mortality [5]. A major association is hypertension and its therapy. There are few evidence-based guidelines for managing these conflicting conditions leading to treatment uncertainty and heterogeneity in clinical practice.

Understanding the underlying pathophysiology of impaired BP regulation is essential for managing it. In this article, we describe healthy BP regulation, ageing-related physiological and pathological changes and the principles behind diagnosis and management.

## Normal BP regulation—Physiology of the baroreflex

BP is regulated by the baroreflex—an automatic, autonomic-mediated reflex responsible for buffering acute changes in BP which may be induced by postural change, exercise, haemorrhage or other physiological stress. The body’s response to orthostasis illustrates this well. When a healthy person stands up, 500–700 ml blood pools into abdominal, pelvic and lower limb blood vasculature. The consequent reduction in venous return to the heart is sensed by stretch-sensitive baroreceptors in the carotid artery and aortic arch, and mechanoreceptors in the heart and lungs. Neural impulses are transmitted to the brainstem (the Nucleus Tractus Solitarius in the pons) via afferent neurons and are integrated and processed in the Central Autonomic Network in the brain—interconnected areas between the brainstem and forebrain responsible for controlling autonomic impulses. Sympathetic nervous system (SNS) outflow is then increased via efferent post-ganglionic autonomic neurons which release noradrenaline. Noradrenaline acts on α- and β-adrenoceptors in the blood vessels (causing vasoconstriction) and heart (increasing heart rate and contractility). In addition, adrenaline is released from the adrenal medulla, and augments β-adrenoceptor activation in the heart, contributing to the increased heart rate and contractility. Finally, the increased sympathetic output also stimulates the hypothalamus to produce anti-diuretic hormone, thus increasing plasma volume and stimulating the renin-angiotensin system [6–9].

These changes occur within seconds, and may manifest as a transient increase in heart rate, a small decrease in systolic BP and rise in diastolic BP [10]. Usually this occurs without symptoms or consequence.

Conversely, when BP rises, baroreceptors and mechanoceptors sense increased stretch and inhibit sympathetic activity. Simultaneously parasympathetic tone is activated via the parasympathetic nervous system’s (PNS) main effector, the vagus nerve. Acetylcholine is released from cardiac ganglia, acting on muscarinic receptors and lowering heart rate, contractility and BP [6, 8, 9].

The two arms of the autonomic nervous system (the SNS and PNS) work together in finely tuned harmony, regulating the BP on a moment-by-moment basis. A healthy system is flexible and responsive, and is capable of reacting to sudden bursts of activity and stress, (mediated by the SNS) and periods of rest and recovery (via the PNS). The reflex arc can be disrupted at multiple points. For example, impairment may occur at the sensing stretch receptors, in the afferent nerves to the brainstem, in the brain and central autonomic network, the efferent pre- and post-ganglionic neurons to the effector organs, the ganglia themselves, or neurotransmitter production and transmission.

## Age-related physiological changes in the baroreflex

The baroreflex—this automatic ability to buffer acute changes in BP by altering autonomic outputs—becomes less sensitive with ageing [9]. This in itself is a risk factor for adverse cardiovascular outcomes [11]. Increasing age results in a reduction in the number, density and sensitivity of β-adrenoceptors in the heart [12]. Paradoxically, and possibly as a compensation, there is increased noradrenaline turnover and decreased reuptake, resulting in higher circulating noradrenaline levels [13–16]. Higher noradrenaline levels correlate with higher resting BP and lower baroreflex sensitivity [14].

Similarly, increasing age is associated with decreased muscarinic receptor density and synaptic density in the PNS [14].

BP variability (another independent cardiac risk factor [17]) increases with age even without an accompanying rise in mean BP [18]. Furthermore, the complexity of this variability—that is, the amount of variability which is organised and structured compared with reactive fluctuations—decreases with age and disease [19]. This decrease correlates with worse heart rate variability parameters [19] illustrating the loss of well-orchestrated baroreflex with age.

There is also a sustained, predictable and linear reduction in the heart’s intrinsic pacemaker rate throughout life [20]. This may be due to reduction in absolute numbers of these highly specialised cells, remodelling, alterations to innervation or change in relative myocyte sensitivity to innervation [20]. As a result, the ability to compensate by reactive tachycardias is attenuated.

## Pathological changes in the cardiac autonomic nervous system and baroreflex

### Cardiovascular autonomic failure

Cardiovascular autonomic failure occurs when there is sympathetic denervation in the heart. This may occur in neurodegeneration or peripheral nerve or ganglionic disorders. Any lesion in the afferent, central or efferent arms of the baroreflex, at adrenoceptors, or interfering with neurotransmitter transmission can be implicated.

‘Cardiac autonomic neuropathy’ is a common end-organ complication of diabetes, with rates as high as 65% in long-standing diabetes [21]. It is an independent predictor of mortality even when corrected for other cardiovascular risk factors. OH (added to other features such as reduced heart rate variability and attenuated nocturnal BP dipping) drives this risk even higher [21]. It also predicts cardiovascular events and progression to kidney disease [21].

Synucleinopathies encompass a spectrum of neurodegenerative disorders involving abnormal protein inclusions of α-synuclein in the brain and autonomic nervous system. The distribution and nature of these inclusions is predictable and stereotyped, and determines the clinical syndrome; for example Parkinson’s disease and Dementia with Lewy Bodies are characterised by Lewy Body inclusions in the basal ganglia and cortex respectively. Multi-System Atrophy involves oligodendroglial cytoplasmic inclusions in pre-ganglionic sympathetic neurons and Pure Autonomic Failure is caused by inclusions in post-ganglionic neurons. These conditions are strongly associated with OH and associated supine hypertension depending on which anatomical correlate of the baroreflex is affected [22, 23]. Neurogenic OH can be identified by a heart rate rise of <15 bpm with OH (revealing inadequate compensation via the baroreflex), whereas non-neurogenic causes with an intact autonomic nervous system (such as dehydration, deconditioning) will have an appropriate heart rate rise of greater than 15 bpm [2].

## Other age-related impairments in BP regulation—Polypharmacy

Medications are the commonest cause of OH-related hospital admission, with multiple medications associated with higher OH risk [24]. A recent meta-analysis found that β-blockers are associated with an odds ratio (OR) of 7.76 for falls, tricyclic antidepressants with OR of 6.3, and α-blockers, antipsychotics and SGLT2 inhibitors also conferring increased risk [25]. No agent for control of hypertension is exempted. Other studies have shown increased risk with Selective Serotonin Reuptake Inhibitors [24] and benzodiazepines [26].

## Other age-related impairments in BP regulation—Deconditioning

Prolonged bedrest results in significant cardiovascular, neural and baroreflex changes. Physiological effects of head down bed rest have been studied extensively as a model for weightlessness in astronauts. Prolonged immobility is associated with reduction in total plasma volume, reduced cardiac filling and stroke volume, and cardiac atrophy [27–29]. There is also reduced lower limb resistance [27] with lower noradrenaline levels and muscle sympathetic nerve activity suggesting SNS withdrawal [30, 31]. Combined with marked lean muscle mass loss seen in older people following bedrest [32], peripheral vasoconstriction to orthostasis is likely to be severely impaired. These changes to the baroreflex can actually modify brain circuitry—detectable changes in the central autonomic areas are seen on functional magnetic resonance imaging after only 24 h of bed rest [33].

## Clinical consequences of the impaired baroreflex

### OH

OH is an adverse prognostic sign for poor outcome across a wide range of conditions: most immediately it is associated with increased risk of falls, syncope and injury [5, 6, 34]. However, it is also associated with cardiac disease including changes in carotid intimal thickness, increased carotid plaque, elevated cardiac injury biomarkers [35] heart failure, coronary artery disease, atrial fibrillation, chronic kidney disease and stroke [36–39]. Ultimately, it is associated with increased death from cardiovascular causes [40] and all-cause mortality [36]. Even in mid-life, its presence is associated with increased dementia risk [41–43] and progression of cognitive impairment to both Alzheimer’s and vascular dementia; higher BP variability is associated with higher risk [43]. As such, it heralds underlying sinister cardiovascular disease and its presence should alert the clinician to address it promptly along with other cardiac risk factors.

### Supine hypertension and OH

Commonly accompanying orthostatic hypotension is neurogenic supine hypertension. This occurs commonly in neurodegenerative conditions affecting the autonomic nervous system. This is defined as supine BP greater than or equal to 140/90 after 3 min [22]. It initially manifests as reduced nocturnal BP dipping, progressing to absence of nocturnal BP dipping, then progressive rise in night-time BP. The association between OH and hypertension is counter-intuitive, but explicable in the context of impaired baroreflex sensitivity to respond to acute changes in BP. The immediate short-term consequence is increased renal perfusion pressure causing increased urinary sodium excretion, and increased diuresis. This results in dehydration the following morning, exacerbating OH [44].

### Essential hypertension and OH

In addition to neurogenic supine hypertension, essential hypertension is another common comorbidity. OH is present in 14–17% of cohorts with hypertension [45–47]. Arterial stiffness, causing hypertension, also affects the baroreflex [48]. Hypertension reduces baroreflex sensitivity which in turn is associated with increased risk of cardiovascular events and death [49]. Higher systolic BPs are associated with greater orthostatic drops [4, 47] and these larger swings conferring increased cardiovascular risk. Thus, it is likely that both conditions are on a spectrum of pathophysiological baroreflex impairment which increases cardiovascular risk.

## Principles of management

Managing OH and concomitant hypertension requires a careful individualised management plan taking into account the immediate challenges of injurious syncope, wellbeing and quality of life (QoL), and longer term cardiovascular and end-organ risks of associated hypertension. There is little evidence in this area, particularly for frail individuals already at high risk of falls and injury, leading to heterogeneity in clinical approach.

It is important to distinguish whether the patient has essential hypertension with OH (from impaired baroreflex), overtreated hypertension or neurogenic OH and supine hypertension from autonomic nervous system disease. If the former, and the patient is otherwise fit, then more intensive BP goals (aiming for systolic BP <120mmHg) may be preferred. More intensive goals are consistently associated with improved cardiovascular outcomes and all-cause mortality without worsening OH [37, 50–52]. It should be noted, however, that the compelling results from randomised control trials are not necessarily generalisable to community older and frailer populations who are at higher risk of falls and OH at baseline than those represented in the trials [53]. Thus, consideration must also be given to the real possibility that overtreatment is being given [54]. To this end, ambulatory BP monitoring is invaluable.

Identifying non-neurogenic causes—for example deconditioning, medications and volume depletion—are critical. Clinicians should understand the distinction in physiology underlying these from neurogenic OH to be able to fully inform their patients. Physical function, risk of injury, emergency department attendance, fear of falling, QoL and time able to stand and walk to an acceptable level should be considered. For patients with advanced neurodegenerative conditions, supportive care to maximise imminent safety and QoL may be of higher priority than longer term cardiovascular goals. Our approach to treatment, incorporating expert consensus guidelines and opinions [2, 10, 55, 56], is summarised below:

***Evaluate trends.*** Home monitoring BP recordings over a range of times and activities can determine trends. A possible schedule could be supine, sitting, and standing BPs early in the morning (before/on getting out of bed), after lunch, at bedtime and when symptomatic. These trends could be taken for a defined period prior to clinic visits or after intervention or medication changes. Twenty-four-hour ambulatory BP monitoring can elucidate this further, and the patient should keep details of meals, alcohol, activity, time getting up and going to bed, and rest periods. It should be emphasised that this is now a ubiquitous and relatively inexpensive investigation. In clinic, an active stand (initial recording after lying supine) for 5 min, then standing BP at 1 and 3 min may confirm OH. If the diagnosis of OH is suspected but not confirmed in clinic, a formal active stand test with BP monitoring beat-to-beat or tilt table testing could be considered.

***Identify and optimise non-neurogenic causes of OH.*** Culprit medications should be reviewed, especially α- and β-blockers, diuretics, nitrates, anticholinergic and psychoactive medications. Night-time anti-hypertensive administration can improve cardiovascular outcomes, with attention to dosage, time of administration and half-life of the chosen medication, which may mitigate effects of supine hypertension and pressure natriuresis [57]. Fluid balance should be assessed, with attention to caffeine and alcohol intake, anxieties about continence and other illnesses such as diarrhoea. Finally, anaemia, vitamin B12 and vitamin D status should be optimised.

***Avoid the supine position completely.*** This should be emphasised to the patient (and carers) explaining the underlying physiology—that this position induces supine hypertension, which leads to pressure natriuresis, dehydration and worsening OH. Similarly, BP augmenting agents must not be taken when supine as they will increase the risk of supine hypertension without manifesting benefits. Patients should be advised to sleep with the head of the bed raised to 12 degrees at least, or higher if tolerated, to avoid this. Steps must be taken to avoid slipping off the bed during the night.

***Avoid bed-rest.*** Hospitalised patients in particular may be considered at risk of falls and unsafe to get up, which will worsen deconditioning effects. Ensuring that the bed is tilted and gradually increasing sitting duration (for example, sitting on the edge of the bed with supervision, in the first instance) should occur as soon as the problem is identified and guided by physiotherapy.

***Ensure fluid repletion***, aiming for 2–2.5 L of fluid per day [56]. Drinking a 500-ml bolus of water prior to standing has a vasopressor effect and is an effective intervention [58]. Compression garments, particularly abdominal binders should be considered [58]. Buttock clenching is particularly valuable in older patients.

***Patients should be reminded to be cautious on standing up***, especially after prolonged resting. Those who are able to should be taught to employ counter-pressure manoeuvres such as squatting, leg crossing and lower body tensing to improve cardiac venous return [59]. Carrying a portable seat can be helpful if standing is unavoidable

***Attention should be paid to size and frequency of meals,*** emphasising avoiding large carbohydrate rich meals and concomitant alcohol, which can cause post-prandial hypotension. If post-prandial hypotension is an issue, it could be used to customise BP control (for example a snack before going to bed, to avoid pressure natriuresis). Patients should also be advised to be cautious about triggers such as hot, warm environments.

***Medications.*** Non-pharmacological management should form the mainstay of treatment, but occasionally pharmacotherapy is indicated.

• Midodrine is a α-agonist, resulting in splanchnic vasoconstriction and increasing BP and it improves BP and QoL in patients with OH and syncope [60–64]. Advantages are a short half-life permitting use when required. Patients should be reminded to avoid the supine position and take the last dose 4 h before bed. Occasional use is preferred to regular use due to tachyphylaxis risk. It should be used in caution with those with peripheral vascular disease, Raynaud’s syndrome or ischaemic heart disease, thyrotoxicosis and phaeochromocytoma.

• Fludrocortisone is a fluid expander which is relatively contraindicated in individuals with supine hypertension as this is a significant risk. It can be effective in OH [65, 66], but is associated with supine hypertension, hypokalaemia and ankle swelling and is poorly tolerated [67].

• Medications which are not yet endorsed in guidelines but which show promise are droxidopa [68, 69], pyridostigmine, octreotide, desmopressin and atomoxetine (which is probably best in antagonising the PNS) [64, 70, 71].

## Managing neurogenic supine hypertension

Avoiding pressure natriuresis by avoiding the supine position is the mainstay of non-pharmacological treatment. In addition, inducing post-prandial hypotension by eating a night-time snack may help avoid pressure natriuresis at night. Short-acting nocturnal agents can be considered, with one consensus guideline suggesting treatment when systolic BP reaches 160–180 mmHg [2]. Short acting nocturnal agents should be preferred over longer acting agents, such as losartan, nitroglycerine patch, captopril, clonidine and hydralazine [2].

## Conclusion

Impaired blood regulation is common in older people, whether due to physiological changes in the baroreflex, age-related diseases or states, or autonomic neuropathies. The consequences—OH and associated hypertension—are debilitating conditions and herald poor prognosis. Identifying the underlying cause, understanding the associated pathophysiology and mitigating exacerbating factors through predominantly lifestyle measures are essential. Future work should focus on identifying optimal BP targets for these individuals, particularly the frailest patients at highest risk of falls and injury.
